# CSAC Characterization and Its Impact on GNSS Clock Augmentation Performance

**DOI:** 10.3390/s17020370

**Published:** 2017-02-14

**Authors:** Enric Fernández, David Calero, M. Eulàlia Parés

**Affiliations:** Centre Tecnològic de Telecomunicacions de Catalunya (CTTC/CERCA), Parc Mediterrani de la Tecnologia (PMT), Building B4, Av. Carl Friedrich Gauss 7, 08860 Castelldefels, Spain

**Keywords:** CSAC, calibration, clock augmentation, GNSS, atomic clock, navigation, steering

## Abstract

Chip Scale Atomic Clocks (CSAC) are recently-developed electronic instruments that, when used together with a Global Navigation Satellite Systems (GNSS) receiver, help improve the performance of GNSS navigation solutions in certain conditions (i.e., low satellite visibility). Current GNSS receivers include a Temperature Compensated Cristal Oscillator (TCXO) clock characterized by a short-term stability (*τ* = 1 s) of 10^−9^ s that leads to an error of 0.3 m in pseudorange measurements. The CSAC can achieve a short-term stability of 2.5 × 10^−12^ s, which implies a range error of 0.075 m, making for an 87.5% improvement over TCXO. Replacing the internal TCXO clock of GNSS receivers with a higher frequency stability clock such as a CSAC oscillator improves the navigation solution in terms of low satellite visibility positioning accuracy, solution availability, signal recovery (holdover), multipath and jamming mitigation and spoofing attack detection. However, CSAC suffers from internal systematic instabilities and errors that should be minimized if optimal performance is desired. Hence, for operating CSAC at its best, the deterministic errors from the CSAC need to be properly modelled. Currently, this modelling is done by determining and predicting the clock frequency stability (i.e., clock bias and bias rate) within the positioning estimation process. The research presented in this paper aims to go a step further, analysing the correlation between temperature and clock stability noise and the impact of its proper modelling in the holdover recovery time and in the positioning performance. Moreover, it shows the potential of fine clock coasting modelling. With the proposed model, an improvement in vertical positioning precision of around 50% with only three satellites can be achieved. Moreover, an increase in the navigation solution availability is also observed, a reduction of holdover recovery time from dozens of seconds to only a few can be achieved.

## 1. Introduction

A new generation of atomic clocks, the Chip Scale Atomic Clock (CSAC), are now commercially available. Atomic clocks price, size and power consumption have been reduced considerably in recent years [1,2]. This tendency is driving Global Navigation Satellite Systems (GNSS) providers to incorporate a CSAC into their high-end products, in particular for Global Positioning Systems (GPS) (i.e., Symmetricom GPS-2700 and GPS-2750) in order to improve the performance of their navigation solutions.

The theoretical basis underpinning GNSS is the triangulation of satellite signals whose range is derived by measuring, as precisely as possible, their propagation time. Thus, clocks are the fundamental components of GNSS; they are used to estimate the propagation time, or equivalently, the pseudoranges [3]. Since the positioning solution performance fully relies on clock measurements, transmitter and receiver clocks must be as synchronized as possible. Currently, the non-synchronization of both clocks is mainly attributable to the accuracy and stability of receiver clocks (which are far less stable than satellite clocks). GNSS receivers normally include a Temperature Compensated Cristal Oscillator (TCXO) clock that exhibits a noisy short-term stability and a poor long-term stability [4]. That is why clock error is estimated epoch by epoch (thus, improving TCXO clocks’ short-term stability) with help from the GNSS signal. To overcome the long-term instability of TCXO, they are time-disciplined with the satellites’ atomic clocks. Although CSAC clocks are also affected by long-term instability, they exhibit high short-term stability [4]. Thus, a combination of CSAC short-term stability and satellite GNSS disciplined long-term stability results in better timing synchronization [5], and better navigation performance (but only if the GNSS signal is not degraded enough to result in an unsatisfactory disciplining process).

A Defense Advanced Research Projects Agency (DARPA) CSAC program was the basis of CSAC technology development [1]. It evolved from classical atomic clocks by reducing their size and power consumption through the use of new technologies such as Micro-Electro-Mechanical Systems (MEMS). The oscillator of atomic clocks uses an electronic transition frequency in the microwave region of the electromagnetic spectrum of atoms. The most common elements used are hydrogen, caesium and rubidium. In the specific case of the CSAC, it is based on caesium. A feedback loop is used to lock the frequency, thus becoming a more stable reference. At start-up, a CSAC’s typical accuracy is 10^−10^ s, improving to 10^−12^ s after 3000 s of GNSS disciplining [6].

Once the CSAC is properly disciplined the GNSS receiver can benefit from its accuracy. First, there is an improvement in the correlator’s synchronism, which is attributable to the reduction of the PLL bandwidth. Thus, it helps improve the tracking recovery time (holdover) and the signal to noise ratio (SNR) [7]. In previous work [6] it has been demonstrated that using a CSAC with a GNSS receiver increases the time to recover after an outage occurs. Other contributions to GNSS navigation are the improvement of the navigation solution in terms of vertical positioning accuracy [4], multipath [7], jamming mitigation and spoofing attack detection [4]. Multipath, jamming and spoofing are well-known GNSS signal degrading effects that are part of the common GNSS vulnerabilities. These vulnerabilities can broadly be related to the system (i.e., receiver), the propagation channel and interferences (accidental or intentional), at which the GNSS community have already proposed many approaches to mitigate their effects [8,9]. A different approach, to improve the signal tracking, to avoid gross pseudorange errors and to mitigate multipath, is the increment of the coherent integration time up to few seconds [10], for which a highly stable clock is required.

Previous studies with CSAC and GNSS receivers show the contribution of atomic clocks to obtain a navigation solution using only three satellites [11,12,13]. In addition, by adding a precise clock to a GNSS receiver, it is possible to obtain position with no need to estimate the receiver clock errors for a long period (<10,000 s) by using the clock coasting estimation method [13]. This technique allows the determination of a positioning solution with only three satellites while classical methods require at least four satellites. The works presented in [12,14] have shown that the combined use of CSAC and GNSS helps mitigate multipath effects. An interesting consequence is that the quality of the navigation solution obtained in non-favourable conditions such as poor satellite availability (<6) or difficult scenarios (urban canyons, and forests) is improved. This means reducing the positioning error. The mitigation of the multipath problem is due to the smaller bandwidth the correlator has to work with, which is a result of the short-term stability of the atomic clock [7].

**Figure 1:**
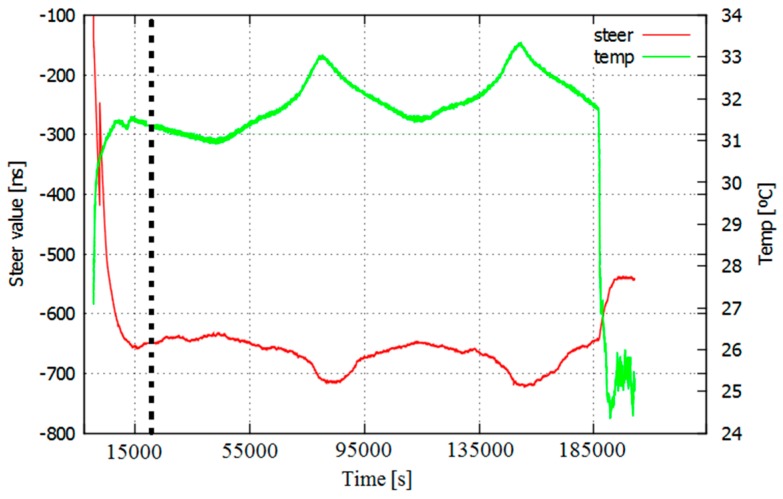
CSAC steering value (red) and room temperature (green), 56 h of data acquisition. The vertical ticked line mark the end of the disciplining process.

However, before integrating an atomic clock into a GNSS receiver, it is necessary to characterize it and model its errors. Details about the CSAC internal elements and some results of its prototyping development and their behaviour are public [1]. CSAC oscillation stability is conditioned by certain factors, such as packaging imperfections. CSAC is built on a thermal isolation package and it self-compensates temperature changes to operate at a constant temperature of around 80 °C [1]. This is similar to how OCXO clocks operate. OCXO are a step beyond TCXO in terms of stability, but their consumption and size are higher due to their intrinsic heating system [15]. However, the lack of an OXCO unit blocked their inclusion on this study. Then, the frequency of the caesium coherent population trapping (CPT) is not perfectly stable, as it deviates from the nominal value. Its resonance frequency deviation is mainly due to changes in the buffer gas and the laser spectrum [1]. In addition, the deterioration of the physical package of the CSAC due to aging also affects the frequency variations (this effect is one of the objects of study). Previous studies show a correlation between the temperature and the steering value of the CSAC (Figure 1) [6,16]. The steering value is a frequency offset correction that affects to the CSAC output frequency.

The main objective of this study is to contribute to the quantification of the benefits of adding a properly modelled CSAC time source to a GNSS receiver. To achieve this objective, the study presented in this paper focus on two complementary purposes. First, it aims to rigorously model CSAC behaviour. To do so, a CSAC clock characterization, calibration (at different temperatures) and aging evaluation were done. The second purpose of the study is to evaluate the impact of the previous characterization and calibration in the terms of track recovery time after outages (holdover), with a properly disciplined CSAC and in terms of position scattering solution, mainly the Vertical Dilution of Precision (VDOP), with a CSAC calibrated by temperature.

The paper is therefore organized as follows: first (Section 2), a brief theoretical overview related to clock modelling and GNSS positioning is presented. After that (Section 3), the research materials and methods needed for the completion of this study are presented. Next, an overview of the expected results is given, (Section 4) followed by the main results of the project (Section 5) and some discussion on them. Finally, the reader will find a concluding section (Section 6), with explanations about the results and some future research directions suggested by the authors of this study.

## 2. Theoretical Overview

Oscillators produce a periodical signal. Far from oscillating at a constant frequency, they always suffer deviations with respect to their nominal frequency [17]. The performance of a clock is normally presented through its stability value, which represents the ability to maintain a nominal frequency output.

In the GNSS context, the following clocks are considered, from high to low performance (in terms of stability):

- Rubidium/Caesium Atomic Clocks—used in satellites;
- Caesium Chip Scale Atomic Clock (CSAC)—used in some high-grade receivers; and
- TCXO—used in most of the commercial receivers.

Although with different severity grades, all oscillators suffer from systematic errors coming from environmental effects such as vibrations, shock, radiation, humidity, temperature and aging. Therefore, by determining these systematic errors it is possible to adjust a better clock model and to improve GNSS performance.

### 2.1. Clock Error Modelling—Allan Variance

**Figure 2:**
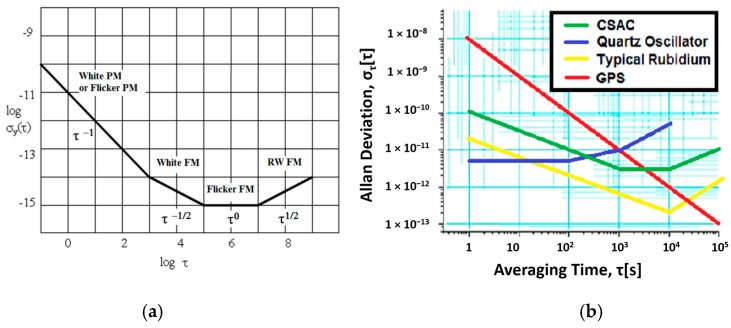
Allan deviation plot: (**a**) a generic plot; and (**b**) a specific one including CSAC and GPS clocks.

The Allan variance (AVAR) [17] is a well-known technique used to measure an oscillator’s stability. This method is suitable for estimating stability due to noise processes (Figure 2), such as the White PM (Phase Modulator) or Flicker PM (related with the quantization noise), the White FM (Frequency Modulator) also known as Angle random walk, the Flicker FM also named BIAS instability and the RW FM (Random Walk Frequency Modulator). The Allan variance is not a tool for the characterization of systematic errors such as temperature effects. Other methods to evaluate clock performance, especially long-term, are theoretical and total variances [17,18,19]. Another different tool, very extended to characterize precise clocks, is the dynamic Allan variance (DAVAR). DAVAR technique is able to obtain a series of common clock anomalies, such as a sinusoidal term, a phase jump, a frequency jump, and a sudden change in the clock noise variance [20]. An advantage of DAVAR compared to AVAR is its ability to model the time-varying stability of a clock [21].

In this study, clock characterization is only based on Allan variance due to our knowledge with this technique, also used to characterize the stochastic noises of inertial sensors (which is another field of work of our research group). Furthermore, the Allan variance is a typical parameter provided by the manufacturers.

The Allan variance, $\sigma_{\gamma }^{2}\left(\tau \right)$, is mathematically defined as:

(1) $$\sigma_{\gamma }^{2}\left(\tau \right)=\frac{1}{2\left(M-1\right)}\;\sum_{\left(i=1\right)}^{\left(M-1\right)}{\left[\gamma \left(i+1\right)-\gamma \left(i\right)\right]}^{2}$$

where *γ*(*ι*) is the normalized frequency difference between the frequency and the nominal frequency averaged over the measurement interval *τ* [17].

In the frequency domain, the Allan variance, $\sigma_{\gamma }^{2}\left(f\right)$, represents the spectral density of the fractional frequency deviation used to identify the different frequency noise sources of oscillators, [17,19]. The fractional frequency is the normalized frequency deviation from its nominal value [16].

(2) $$\sigma_{\gamma }^{2}\left(f\right)=\;h_{-2}f^{-2}+h_{-1}f^{-1}+h_{0}+h_{1}f+h_{2}f^{2}$$

where *f* is the sampling frequency and $\sigma_{\gamma }^{2}\left(f\right)$ is the clock’s variance power spectrum. Typically, only h_0_ (white noise), *h*_−1_ (flicker noise), and *h*_−2_ (random walk noise) values are used to define the clock’s Allan variance and to model the clock error behaviour. The theoretical noise values of CSAC and TCXO clocks are obtained from Figure 2b [22] and their values are shown in Table 1 (these values are generic for a certain unknown conditions and provided by the CSAC manufacturer).

### 2.2. Clock Steering and Disciplining

All clocks have an inherent drift, which is not constant in the long term. The process considered in this study consists on estimating and applying an oscillator frequency correction parameter: the steering value. In order to obtain this value, the oscillator under evaluation is monitored and compared with a reference oscillator by measuring the phase difference between both clocks [13,23]. This measurement can be performed through a disciplining process.

Disciplining is the procedure of computing the steering value and using it to modify the oscillation frequency. To obtain a reliable steering value, the previously presented procedure is periodically carried out, computing clocks phase difference between CSAC and a PPS (Pulse Per Second) event generated by the GNSS receiver. The obtained steering values are time-filtered to avoid errors due to jitter noise. After the completion of the disciplining process the clock achieves its maximum stability. The accuracy of the final computed steering value depends on many factors such as aging, environment (i.e., temperature, magnetic fields, etc.) and reference clock performance. For proper clock performance, the estimated steering value should be applied in every clock initialization. However, this value is not constant over time, and it must be periodically recomputed. The variability of the CSAC steering value is presented in the next section.

A critical step in the disciplining process is the selection of the data averaging time. A joint analysis of target clock error models and reference clock error models must be performed. This can be easily done by means of the Allan Variance charts: the optimal averaging time is that at which the noise of the reference clock and the noise of the target clock are the same. If the averaging time is lower than the intersection clock noise values, the algorithm will not reach the maximum stability due to the poor short-term stability of the reference oscillator. Otherwise, if the selected averaging time is higher than the intersected value, the algorithm will take more time to reach the stability. If it is wished to discipline a GNSS receiver, the data of Figure 2b can be used. According to this, the GNSS and CSAC noises are coincident at approximately *τ* = 3000 s. Other studies from the manufacturer specify that it is better to set *τ* = 5000 s [24]. To obtain an accurate steering value the disciplining time should be at least 3 times the disciplining filter window. Therefore, this process usually requires few hours (i.e., with a filter window of 5000 s, the process will take from 3 to 4 h).

### 2.3. GNSS Position and Clock Correction Estimation

A brief review of the code-based position and clock correction estimation algorithm is presented in this section. One of the methods that GNSS receivers implement to estimate the position and clock correction value is through the pseudorange measurements, that is, the distance between the satellites and the receiver. In order to measure these pseudoranges the GNSS receiver computes the time that the signal takes to travel from the satellite to the receiver. As an initial approximation, if we do not consider the atmospheric (ionospheric and tropospheric) refractivity variations, it is possible to obtain the distance between the GNSS receiver and the satellite by using the speed of light constant. Finally, knowing the satellite positions, the receiver localization is obtained by means of triangulation. Since the clock receiver is not perfectly synchronized with the satellite’s clocks, the error in measuring travel times is directly translated into a systematic error in pseudoranges. This error can be up to 0.3 m for each pseudorange measurement, leading to a fully incorrect position estimation (few meters of error). The value of 0.3 m is obtained by the multiplication of the theoretical TCXO clock bias (10^−9^) by the constant of light velocity [25].

Thus, GNSS receivers usually require at least four pseudoranges to solve the receiver’s position and clock correction. The system needs to determine four unknowns; three are to determine the position and the last is to determine the receiver’s clock error. The following equations must be completed for each satellite in a single GNSS signal frequency processing (i.e., GPS L1):

(3) $$P^{k}+v^{k}=\|X-X^{k}\|+c\cdot \left(b+f\cdot \Delta t\right)+K^{k}$$

$$X^{\prime }=0+w_{X};\;\sigma =\sigma_{d}$$

$$b^{\prime }=f+w_{b};\;\sigma =10^{-9}$$

$$f^{\prime }=0+w_{f};\;\sigma =10^{-10}$$

where *P^k^* is the observed pseudorange, *v^k^* is the residual error, *X^k^* is the known satellite position, *K^k^* are the atmospheric and instrumental corrections, and *X* and (*b*, *f*) are the unknown receiver position and the unknown receiver clock corrections (bias and drift). The drift is multiplied by a time increment (∆*t*) and *c* is the constant of light velocity. The receiver position is considered as a random walk process ($X^{\prime }$) with its process noise ($w_{X}$) directly related to the vehicle dynamics (linear and angular velocities) of around $\sigma_{d}$, while the clock error is considered as a stochastic process controlled by b and f with a bias process noise ($w_{b}$), of around 10^−9^ (Allan deviation at *τ* equal to 1 s) and a drift process noise ($w_{f}$) of around 10^−10^. Please note that this system is not observable for less than four pseudoranges.

By using a stable oscillator, such as the CSAC (with a known clock model error), it is possible to estimate the position with only three satellites. This can be achieved for a period (typ. < 10,000 s) by synchronizing the CSAC with the GNSS time. Due to the short-term stability performance, the CSAC can be considered as a reference clock value whose residual error is below 10^−12^ s, or equivalently below one millimetre. Thus, it can be assumed that the system is only able to determine a solution within specifications with three satellites. The following equations must be completed for each satellite and each available frequency:

(4) $$P^{k}+v^{k}=\|X-X^{k}\|+c\cdot dt+K^{k}$$

$$X^{\prime }=0+w_{X};\;\sigma =\sigma_{d}$$

$$dt^{\prime }=0+w_{t};\;\sigma =10^{-12}$$

where *P^k^* is the measured pseudorange, *X^k^* is the known satellite position, *K^k^* are the modelled or provided atmospheric and instrumental corrections, *dt* is the receiver clock correction, *X* are the unknown receiver position and *c* is the constant of light velocity. *v^k^* is the residual error.

In the new model, the receiver position is once again considered as a random walk process with a process noise ($w_{X}$) directly related to the vehicle dynamics, while the clock error is considered as a random walk process with a process noise ($w_{t}$) of around 10^−12^ [26].

## 3. Research Materials and Methods

### 3.1. Materials

**Figure 3:**
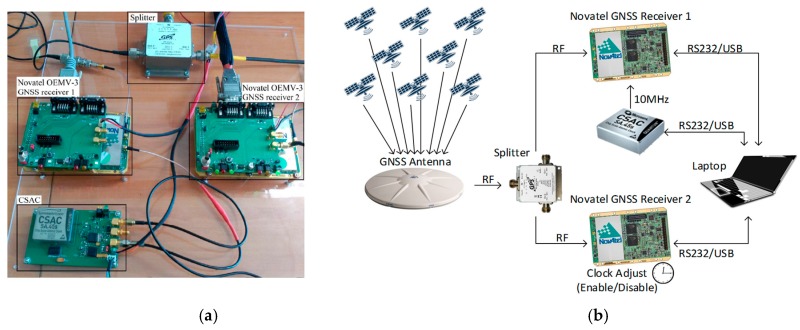
Basic CSAC configuration (**a**); and CSAC clock characterization example setup (**b**).

To carry out this study, a CSAC SA.45s from Microsemi and two survey-grade GNSS Novatel OEMV-3 receivers with a shared L1/L2 antenna were used. To study and quantify the improvement of the navigation through the addition of an atomic clock, two identical GNSS receivers (sharing the antenna), and only one with an atomic clock, were compared. Both receivers, when operating together, receive the same GNSS signal from a high-grade splitter (S12 from GPS SOURCE manufacturer, Pueblo, CO, USA). All of these elements can be easily identified in Figure 3.

The configuration of the CSAC changes depending on the type of test to run. For example, in some scenarios the CSAC is evaluated after a disciplining (thus, using a PPS input,) while in other scenarios, it is evaluated in a free running mode after setting up a steering value. More details on CSAC configuration commands can be found in [6,16].

In the same way, the Novatel GNSS receiver is configured to better adapt its behaviour to the type of test being performed. The main configuration options and log commands used are clock model data logging and external clock enabling/disabling.

### 3.2. Methods

A rigorous procedure must be implemented [6,16] to properly characterize and calibrate the CSAC. The characterization starts with an estimation of the delays introduced by the system due to cables and electronic components. Later on, a disciplining of the CSAC and an initial evaluation of its performance (in terms of oscillation stability) are carried out. According to the CSAC datasheet provided by its manufacturer, the maximum fractional frequency change over an operating temperature range (from −10 °C to 35 °C) is 5 × 10^−10^. Since our evaluation corroborates that the estimated steering value is strongly correlated with temperature (Figure 1), the characterization process must take the temperature coefficient into account. To do so, the previous calibration process is repeated for a preselected range of temperatures within a climate chamber [16]. The climate chamber is configured to reach a stable temperature (or set of temperatures) for a long time period (2–4 h) while the devices under evaluation are placed inside it. From that, a temperature-dependent calibration function is obtained. This function relates the temperature read by the CSAC and the steering value to be applied to the CSAC to maximize its performance.

In addition, the CSAC calibration values are not constant in time due to CSAC degradation. The aging effect of a CSAC specified by its manufacturer is approximately about 9 × 10^−9^/month. In the frame of this study, the previous temperature-dependent calibration procedure is repeated once a month for 4 months.

Once the system is properly modelled, the study focuses on the analysis of the new models in holdover recovery and positioning performance.

#### 3.2.1. Procedure for CSAC-Related Elements and Interface Board Characterization

**Figure 4:**
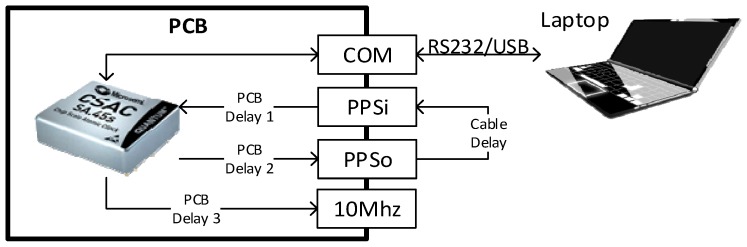
Basic CSAC configuration, measuring delays test configuration.

This test aimed at separately characterizing CSAC-related elements delays. Cables, the electrical components and microstrip lines in the Printed Circuit Board (PCB), or interface board, introduce some delays that have to be taken into account. Figure 4 shows a scheme depicting the different delays. Once those delays are measured, it is possible to synchronize the CSAC with the Novatel GNSS receiver.

The internal CSAC phase meter tool is used to measure cable delays. All cables used comply with the Conducfil RG-174/U 50 Ohm MIL C-17 standard. The manufacturer [6] specifies a propagation speed of 66% of light in the vacuum (3 × 10^−8^ m/s).

**Figure 5:**
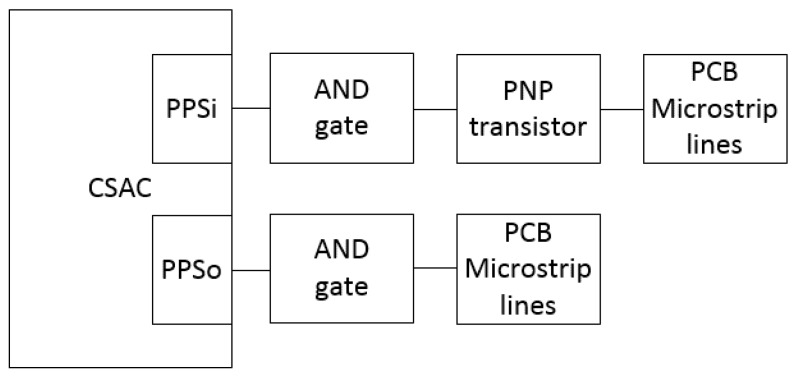
PPSi and PPSo blocks diagram representing the main delays sources on the CSAC PCB.

An oscilloscope and a compensated probe are used to measure the delays in the PCB. Figure 5 presents the main elements producing delays in the PCB. The threshold to decide that a change in a signal occurred is a variation of at least 10% of its maximum value; for instance, in PPS signals the signal edge is considered when the signal reaches 0.33 V, since their maximum value is 3.3 V. Theoretical values of AND gate and PNP transistor delays can be obtained from the datasheets provided by each manufacturer and the PCB lines (microstrip) delay is calculated by means of Equation (5), where *τ* is the delay in the PCB line, *τ*_0_ (equal to 55 ps/mm) is a generic delay for FR-4 grade microstrip lines [6] and *λ* is the microstrip line length:

*τ* = *τ*_0_·λ (5)

#### 3.2.2. Procedure for CSAC Temperature Calibration

To analyse the oscillator frequency response in relation to temperature, the first step is to discipline the CSAC. This is done using a GNSS receiver in a controlled temperature environment achieved by means of a climate chamber (CC). Several tests are required at different temperatures. The temperature ranges to consider are from 5 °C to 40 °C. The CSAC is set on disciplining mode (filter window = 5000 s) using the PPS signal from the GNSS (using its internal TXCO) receiver set as an input; the monitored parameters are the steering and phase meter values.

The output of this procedure is a series of temperature and steering related values within the tested range. With the data series, an equation is obtained to interpolate the values between each temperature step. This equation is used to calibrate the CSAC. The calibration consists of measuring the environmental temperature and setting up a CSAC steering adjustment value. Any change in temperature implies a change in the steering value applied to the CSAC. This is very important when working in dynamic environments, where the air/room temperature cannot be kept constant.

#### 3.2.3. Procedure for Holdover Analysis

**Figure 6:**
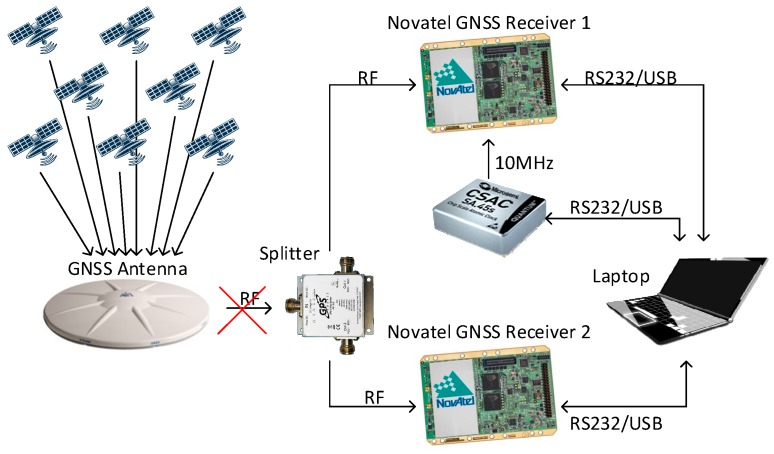
CSAC holdover measurements configuration.

This test intended to analyse the performance of the GNSS receivers when an outage occurs. Its purpose is to observe how the clock stability affects the receiver’s capability to re-track the satellites. To do this, the time elapsed between the moments when the signal is recovered and the receiver tracks the satellites again is measured. As in the previous tests, one of the GNSS receivers used its internal clock while the other one used the CSAC as an external clock (see Figure 6). Different outage intervals were tested to evaluate how the clocks’ drift—a phenomenon that increases with time—affects the time needed to re-track the satellites.

#### 3.2.4. Procedure for Positioning Analysis

Once the CSAC is modelled by temperature, three test scenarios are considered in order to evaluate the benefits on the positioning performance:

- A GNSS receiver using its internal TCXO;
- A GNSS receiver using an external CSAC, without temperature compensation; and
- A GNSS receiver using an external CSAC, with temperature compensation.

For each of the scenarios the same conditions are used. The positioning solutions are cross-compared, evaluating the performance of the CSAC by applying the previously mentioned temperature calibration procedure. The lack of an independent reference position limited the evaluation of these results to their precision and not to their accuracy.

## 4. Expected Impact of Improving CSAC Calibration in Holdover and Positioning Solution

Due to its high short-term stability, it is expected that the availability of the positioning solution will improve when a holdover happens. The drift of the CSAC clock with respect to the satellite clock must be some orders of magnitude lower than the drift of the TCXO stand-alone (without the GNSS corrections). Therefore, the ability of the GNSS receiver to more quickly recover the positioning solution must be easily noticeable when comparing it to a traditional GNSS receiver (with an internal TCXO). The receiver manufacturer specified a Time to First Fix (TTF) for a hot start of 35 s and a signal reacquisition for L1 of 0.5 s (typical values).

In terms of positioning accuracy, the vertical or height component (VDOP) should benefit the most from this CSAC augmentation configuration. This is because the vertical component has a strong correlation with the clock performance. No improvements are expected in planimetry. The use of atomic clocks as CSAC not only makes it possible to work with fewer observations (satellites) but also has an impact on the solution performance. With this equipment, the modelling of the clock parameter is simpler and more reliable, leading to a more accurate estimation. This improvement in the clock accuracy has an impact on the accuracy of the position unknowns. There are several studies [4,5,12,13] that demonstrate that this improvement has a different effect on each computed parameter.

## 5. Results and Discussion

### 5.1. CSAC Environment Characterization

The first step of the process is to collect the theoretical (reference) values of the system delay, which are shown in Table 2. The delay in the PPS_i_ signal is mainly caused by the copper lines in the PCB, the PNP transistor and the AND logic gate placed between the SMA connector and the CSAC input pin (see Figure 5). In the case of the AND gate, the lowest typical value (5 ns) is higher than the one measured in the lab (3.52 ns). Therefore, the measured value is used as the reference (the difference can be due to manufacturing tolerances). The PNP transistor datasheet defines a delay of 10 ns for a 60 V change. Hence, the delay for a 3.3 V change can be extrapolated to 0.55 ns. Finally, Equation (5) is used to calculate the PCB microstrip line delay.

**Figure 7:**
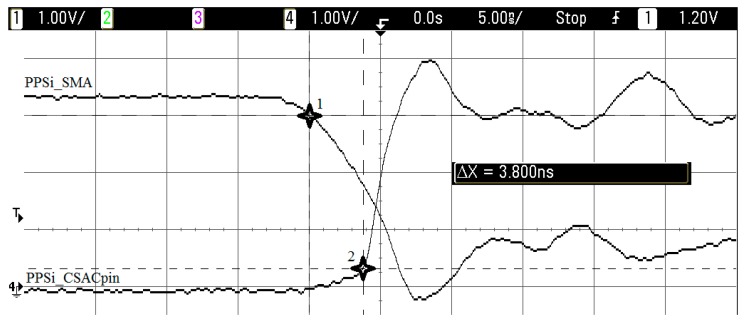
PPS_i_ signals delay (example). The horizontal scale is one volt and the vertical scale is five nanoseconds per division, respectively.

Next, the obtained measurements were performed using an oscilloscope probe. A print screen of these measurements can be found in Figure 7, and Table 2 and Table 3 show the collected figures. In particular, Figure 7 shows the measurement of the first row of Table 3.

After evaluating the phase meter measurements of cable delays, an inconsistence between these measurements and the theoretical expected values was observed. All the measured values were significantly lower than expected (having a negative offset). We assume that this offset value is due to a time delay compensation stored in the CSAC internal memory set prior to shipment (from factory). Therefore, the measured values for cable delays shown in Table 3 are corrected with the addition of a negative offset, with a value of −3.16 ns, computed using a linear regression modelling.

### 5.2. Temperature Calibration Performance

Before starting the calibration procedure, it is necessary to analyse the reliability of the temperature sensor of the CSAC. Table 4 shows the relation between the temperature inside the Climatic Chamber (T_CC_) and the temperature measured by the CSAC (T_CSAC_). It can be observed that there is a temperature bias of around 1.5 °C. This bias needs to be taken into account when assigning steering values related to environmental temperature.

#### 5.2.1. Clock Stability Analysis

**Figure 8:**
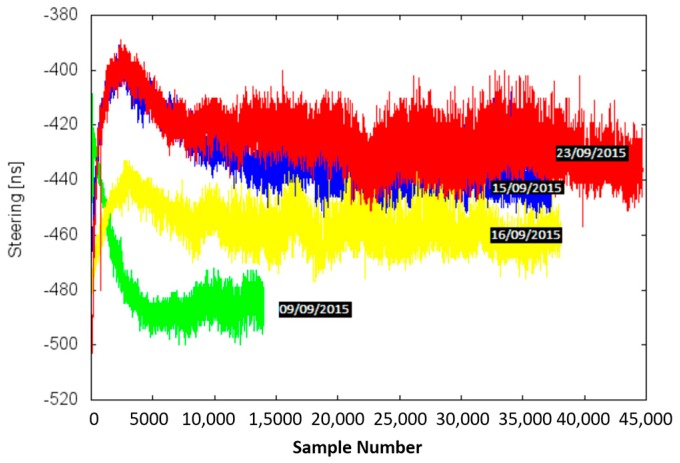
CSAC time drift evaluation.

Initially, several tests at the same temperature are carried out inside the CC in order to observe the reliability of the steering values once the procedure converged. The computed values are plotted in Figure 8. A slight drift, and a smaller random variability effect on the obtained steering parameter (jitter) are observed. This random variability effect is observed in the third test (16th of September, yellow line in Figure 8). From these results, the final steering value generated is based on the mean value of the performed tests. The mean value is calculated from all the samples where the phase meter measurement is equal to 0 ns.

Another effect observed from these tests, done with a time-lapse of three weeks, is that there is an aging drift in the data. This effect is evaluated more in detail in Section 5.2.2. Due to this time drift, a calibration temperature must be performed in the shortest possible period.

**Figure 9:**
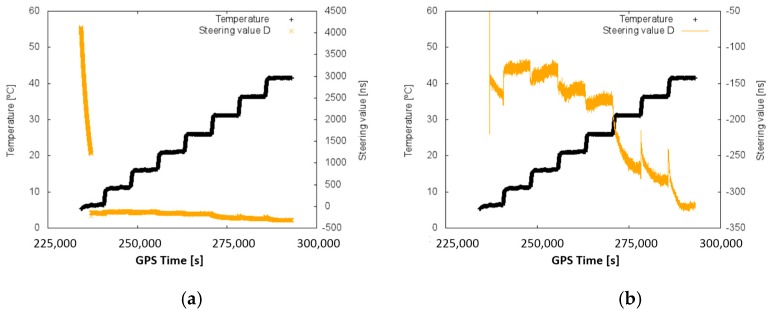
Steering and temperature vs. time (**a**). Steering values zoomed only in the right-side vertical axis (**b**). Temperature and time axes are constant in both cases.

Once the determination of the steering values is performed, a set of acquisition campaigns was carried out at different temperatures. Figure 9 presents the output of a disciplining process at different temperatures (since the initial steering values are high, a zoom of these results when the system converges is also presented). Hence, in Figure 9b, the most stable part is zoomed (minimum slope).

In the previous figure, the big changes in the measured steering parameter that are produced by a quick change in temperature (temperature steps) are easily observable.

From that campaign, a second order equation relating steering values and temperature is derived (Equation (5)). The values shown in the equation were computed from tests similar to the one shown in Figure 9. In the equation, the steering adjustment parameter (s), expressed in ns, depends only on the temperature measured (T_CSAC_), expressed in degrees Celsius, by the CSAC.

s = −0.164920 × T_CSAC_^2^ + 3.977709 × T_CSAC_ − 178.069972 (5)

It is worth mentioning that the initial steering values for the disciplining CSAC are very noisy because the algorithm has not reached the stable stage (left side of left plot in Figure 9). This phenomenon is always present in the disciplining mode. The only way to reduce it is by setting the best possible initial value.

**Figure 10:**
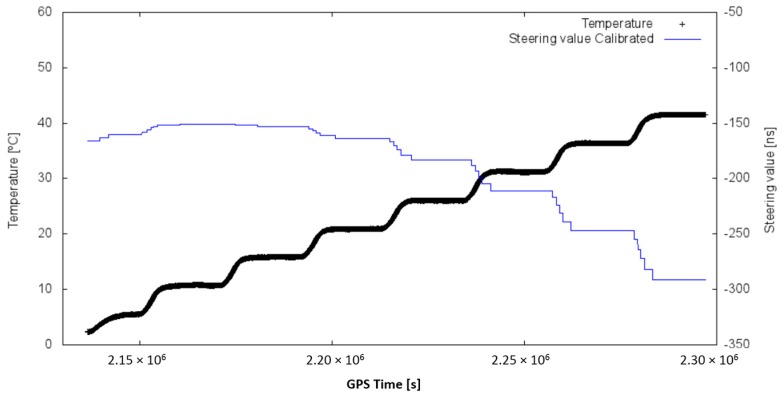
Steering and temperature vs. time.

Finally, to validate the previous values, another test using the estimated steering values as initial values in the disciplining process was carried out (Figure 10). Now, it can be seen that the convergence time is close to zero, which means that the initial steering values are correct.

#### 5.2.2. Aging

To evaluate the effect of frequency deviations due to the aging of the CSAC, the same temperature compensation procedure is repeated during four months with a time lapse of 3–4 weeks. Table 5 shows the results of the obtained steering values at 25 °C.

The mean and standard deviation results obtained from the steering values at 25 °C are presented in Table 6.

**Figure 11:**
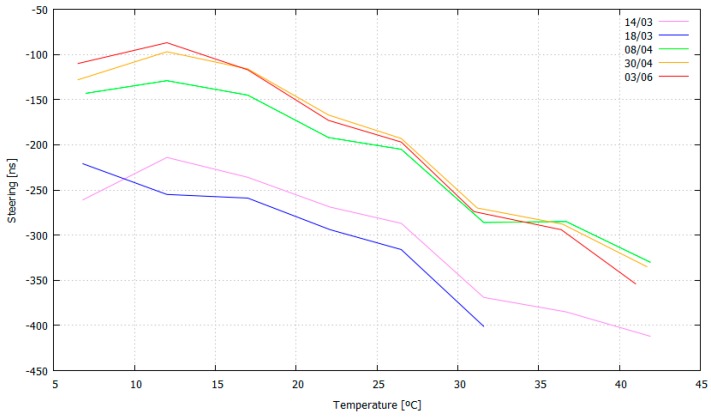
Steering values (*y*-axis) vs. temperature (*x*-axis) on long term (coloured labels).

Observing the data in Table 5, the aging effect and its random nature can be observed. Thus, an aging effect on the CSAC is clearly observed, and can be more deeply evaluated in Figure 11, which includes measurements at different temperatures.

From the previous plot, the time drift of the previous values obtained (after three months) is clearly observed. Henceforth, the last obtained values (03/06) were those used in the following tests.

### 5.3. Holdover

**Figure 12:**
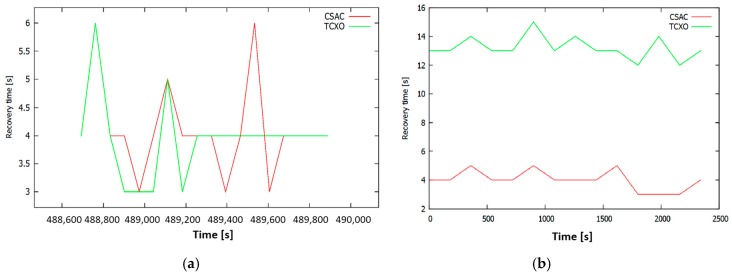
CSAC and TCXO holdover of: 10 s (**a**); and 120 s (**b**).

Several tests were performed to check the recovery time varying the duration of the signal outage. Figure 12a shows the results of the 10-s outage test. In this case, the contribution of the CSAC is negligible, since no improvement in the time to re-track was observed. On some occasions, the TCXO is faster to re-track, but in a number of other cases the opposite situation is common.

The results obtained in the 1-min test show that the use of the CSAC improves the recovery time. Please note that a case was found in which the TCXO behaved better than its nominal value. When increasing outages to 2 min (Figure 12b), it is observed that the time needed by the CSAC to recover the lock is about three times shorter. In the 5-min tests, TCXO reached its maximum mean value of 15 s. A summary of time recovery after outages is shown in Table 7.

The conclusions drawn from the tests above indicate that no improvements in the recovery time may be expected due to the use of the CSAC for signal outages shorter than 1 min. Beyond this time lapse, the CSAC clearly improves the time needed to re-track. Moreover, the time needed to recover the lock when using the CSAC is not affected by the duration of the outage; in contrast, it increases when using a TCXO. In all cases, the recovery time is below the TTF hot start manufacturer specifications (typically 35 s).

### 5.4. Positioning

#### 5.4.1. Altitude Jitter (and VDOP)

**Figure 13:**
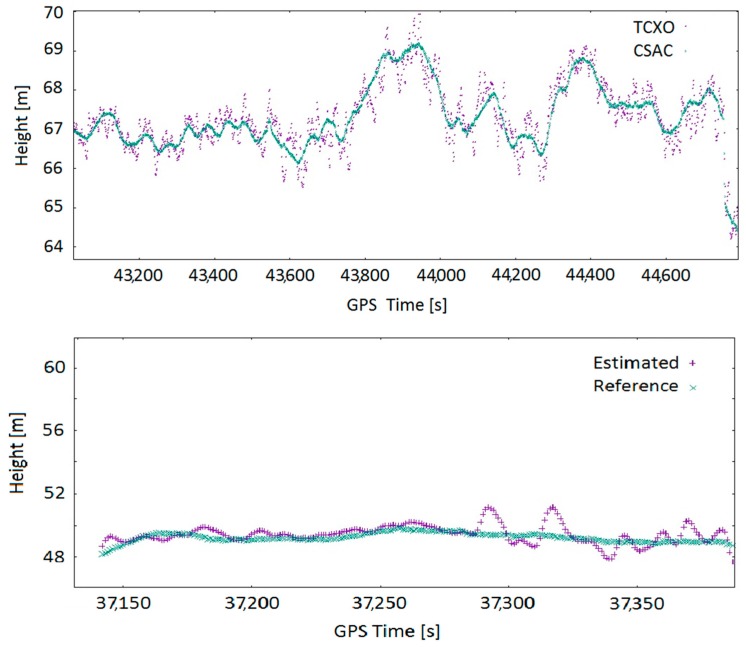
Height estimation for: static (**top**); and dynamic (**bottom**) acquisitions.

In Figure 13, small windows of the height estimation for a static and a dynamic acquisition are presented. The dynamic test was performed using a car and the trajectory consists on making several oval rounds inside a parking lot. The purple samples represent the estimated solution of the equipment using TCXO while the green samples represent the solution including the CSAC clock. The mean of a windowed standard deviation is presented in Table 8. These values do represent a significant improvement in the height precision determination; specifically, using a properly-modelled CSAC reduces standard deviation by a factor of 2.

#### 5.4.2. Scattering and Planimetry

**Figure 14:**
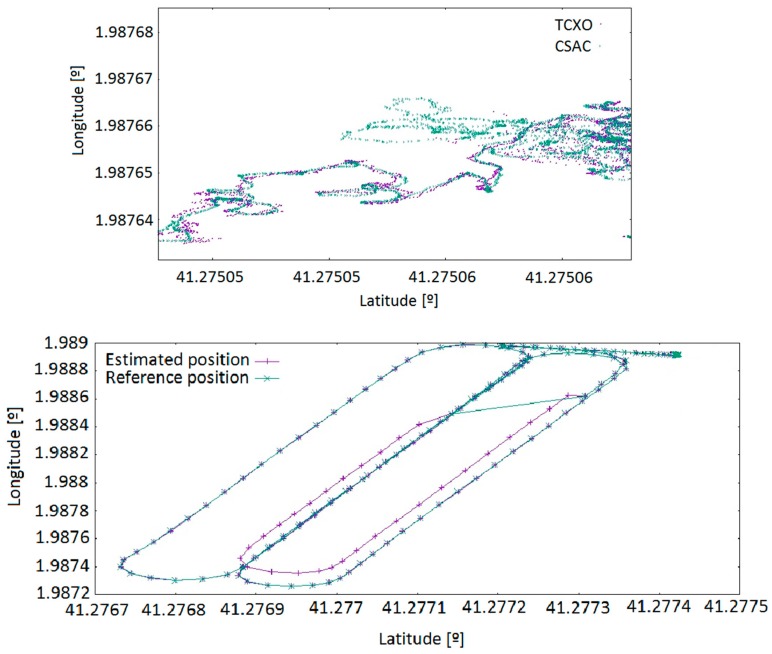
Available positions for: static test (**top**); and dynamic test (**bottom**).

Figure 14 offers another view of the outputs of the navigation algorithm for the equipment working with TCXO and the equipment working with CSAC in static and dynamic environments. Both tests have been processed with a variable number of available satellites, from three satellites up to ten satellites. As long as there are enough available satellites, both solutions are quite similar. However, when the number of satellites decreases to three, the system using the CSAC clock is still able to provide a solution within the GNSS specifications, while the equipment with TCXO cannot. Table 9 presents the mean of the standard deviation of all static tests using each of the available equipment. From these results, no relevant improvement in terms of planimetric positioning can be deduced. It also presents the mean of the standard deviation of the difference between reference trajectories and target trajectories. Please note that the slots with three satellites have not been taken into account. Then, the reference trajectory is coincident with the CSAC trajectory. No relevant improvement in planimetric positioning can be deduced from this result.

#### 5.4.3. Clock Error Estimation

**Figure 15:**
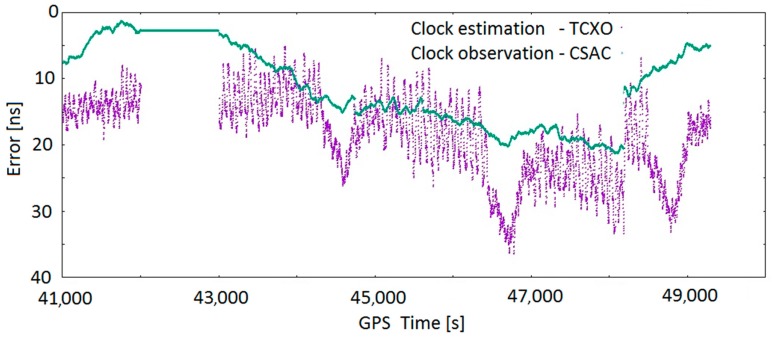
TCXO clock correction estimation and CSAC clock correction observation.

Figure 15 presents both the error-estimated correction for the TCXO error (purple) and the error-estimated correction for the CSAC error (green). As can be seen, both the magnitude and the shape of the two errors are quite different. In the three-satellite period, a gap in the TCXO clock estimation is observed due to the insufficient number of satellites, while the CSAC clock maintains the clock observation. As can be observed, the variability of the TCXO correction is some orders of magnitude higher than that for CSAC. Thus, it can be deduced that the impact in the solution of an incorrectly estimated TCXO error will be higher than in the case of CSAC.

## 6. Conclusions

This study presents a characterization of the CSAC, examining in detail the delays introduced by the interface (PCB) board for a better synchronization of CSAC and a GNSS receiver. The measured delay values are slightly different from the theoretical values but these minor differences could be due to the tolerances of the components used and some approximations of the values (i.e., cable delays). It is also unknown whether the CSAC internally corrects the phase meter measurements, though the results seem to prove that it does. In any event, these results are considered consistent with the expectations.

The temperature calibration process of the CSAC is proven as valid. The clock performance of the CSAC is greatly improved and the system is able to work in a dynamically changing environment, keeping the clock stability to its lowest possible value (10^−12^, see Figure 9). Instead, the traditional disciplining process (of some hours of duration) cannot compensate changes in the room temperature. The aging of the CSAC has been proven to coincide with the manufacturer’s specifications (9 × 10^−9^ s/month) in most of the cases, but not always (i.e., data series 18/03 and 08/04 in Figure 11). This could be due to a degradation of the packaging, which would be less constant than expected over time. More evaluations in the aging of the CSAC can help to determine the causes of its degradation irregularity.

In the particular case of the holdover performance evaluation, everything worked as expected (see Table 7). For short holdover time lapses (10 s), there are no differences between the CSAC and the TCXO results. The holdover time lapse is so short that neither clock has degraded enough to affect the recovery time of the receiver. In another evaluated case, with a higher time lapse (1 min or more), the CSAC significantly helped stabilize the recovery time needed (no more than 4 s, see Figure 7). On the other hand, in the case of the TCXO, this time was increased, and had a random behaviour. Hence, the recovery behaviour of a GNSS receiver, with a CSAC integrated, is always equal or better in a hot start condition than one with its internal TCXO.

It was proved that the CSAC clock makes it possible to estimate position within specifications even when only three satellites are available. It has also been observed that the impact of reducing and simplifying the clock unknown has almost no impact in planimetry performance neither for static nor dynamic trajectories. However, the results demonstrate that having access to these precise clocks has a relevant impact on the performance of height estimations in both scenarios.

Future research will focus on the study of the benefits of the use of a CSAC in jamming and spoofing scenarios. Moreover, the hot/warm/cold start behaviours in different scenarios can be more deeply evaluated.

## Figures and Tables

**Table 1:** Theoretical clock error modelling, TCXO and CSAC.

Clock Model	White Noise (*h*_0_)	Flicker Noise (*h*_−1_)	Random Walk Noise (*h*_−2_)
TCXO	9.4 × 10^−20^	1.8 × 10^−19^	3.8 × 10^−21^
CSAC	7.2 × 10^−21^	2.6 × 10^−23^	2.7 × 10^−27^

**Table 2:** Theoretical PPS_i_ and PPS_o_ delays, but AND gate delay is obtained by and oscilloscope measurement.

Component	Delay (ns)	
	PPS_i_	PPS_o_
AND gate	3.52	3.52
PNP transistor	0.55	-
PCB microstrip line	0.40	0.40
**Total**	**4.47**	**3.92**

**Table 3:** Subsystems measured delays.

Component	Delay (ns)		
	Expected	Measured by	
		Oscilloscope	Phase Meter *
PPSi PCB	4.47	**3.80**	-
PPSo PCB	3.92	**3.76**	-
Cable A (length = 0.7 m)	3.53	-	**3.89**
Cable B (length = 1.4 m)	7.17	-	**6.65**
Cable C (length = 2.1 m)	10.60	-	**10.76**

* Measured values are corrected by adding an offset correction value of 3.16 ns.

**Table 4:** CC temperature measurements.

Temperatures (°C)	
T_CC_	T_CSAC_
40 ± 0.09	41.55 ± 0.05
25 ± 0.09	26.40 ± 0.05
5 ± 0.09	6.60 ± 0.25

**Table 5:** Obtained steering values at 25 °C.

Date	Steering Value at 25 °C
14 March	−287 ns
18 March	−316 ns
8 April	−205 ns
30 April	−193 ns
3 June	−197 ns

**Table 6:** Calculated steering values statistics at 25 °C.

Parameter	Steering Value at 25 °C
Mean	−239 ns
Std. dev. (σ)	58 ns

**Table 7:** Holdover values.

		Holdover Outage Time (s)			
		10	60	120	300
Recovery time (mean) [s]	CSAC	4	4	4	4
	TCXO	4	13	13	15
Recovery time (SDEV) [s]	CSAC	1	1	1	1
	TCXO	1	4	2	1

**Table 8:** Static and dynamic tests—height error standard deviation.

	Standard Deviation	
	Static Tests	Dynamic Tests
TCXO	50.0 cm	55.0 cm
CSAC	25.5 cm	24.3 cm

**Table 9:** Static and dynamic tests—planimetric error and mean standard deviation taking a single point as a reference in the static test and an external reference trajectory in the dynamic test.

	Mean Standard Deviation	
	Static Test	Dynamic Test
TCXO	40.0 cm	0.1 cm
CSAC	38.5 cm	0.0 cm
